# Success rates, challenges and troubleshooting of left bundle branch area pacing as a cardiac resynchronization therapy for treating patients with heart failure

**DOI:** 10.3389/fcvm.2022.1062372

**Published:** 2023-01-10

**Authors:** Junmeng Zhang, Yimin Zhang, Yaxun Sun, Mengna Chen, Zefeng Wang, Changsheng Ma

**Affiliations:** ^1^Department of Cardiology, Beijing Huaxin Hospital, Tsinghua University, Beijing, China; ^2^Department of Cardiology, Sir Run Run Shaw Hospital, Zhejiang University School of Medicine, Hangzhou, China; ^3^Department of Cardiology, Beijing Anzhen Hospital, Capital Medical University, Beijing, China

**Keywords:** left bundle branch area pacing, heart failure, cardiac resynchronization therapy, success rates, troubleshooting

## Abstract

Cardiac resynchronization therapy (CRT) is an important treatment of heart failure patients with reduced left ventricular ejection fraction (LVEF) and asynchrony of cardiac electromechanical activity. Left bundle branch area pacing (LBBaP) is a novel physiological pacing modality that appears to be an effective method for CRT. LBBaP has several advantages over the traditional biventricular-CRT (BiV-CRT), including a low and stable pacing capture threshold, a high success rate of implantation, a short learning curve, and high economic feasibility. However, LBBaP is not suitable for all heart failure patients needing a CRT and the success rates of LBBaP in heart failure patients is lower because of myocardial fibrosis, non-specific intraventricular conduction disturbance (IVCD), enlargement of the right atrium or right ventricle, etc. In this literature review, we summarize the success rates, challenges, and troubleshooting of LBBaP in heart failure patients needing a CRT.

## Introduction

Cardiac resynchronization therapy (CRT) is an important treatment of heart failure patients with reduced left ventricular ejection fraction (LVEF) and asynchrony of cardiac electromechanical activity (1). The strategies for achieving cardiac resynchronization include biventricular-CRT (BiV-CRT) and physiological pacing. His bundle pacing (HBP) and left bundle branch area pacing (LBBaP) are both physiological pacing techniques (2, 3).

**Figure 1:**
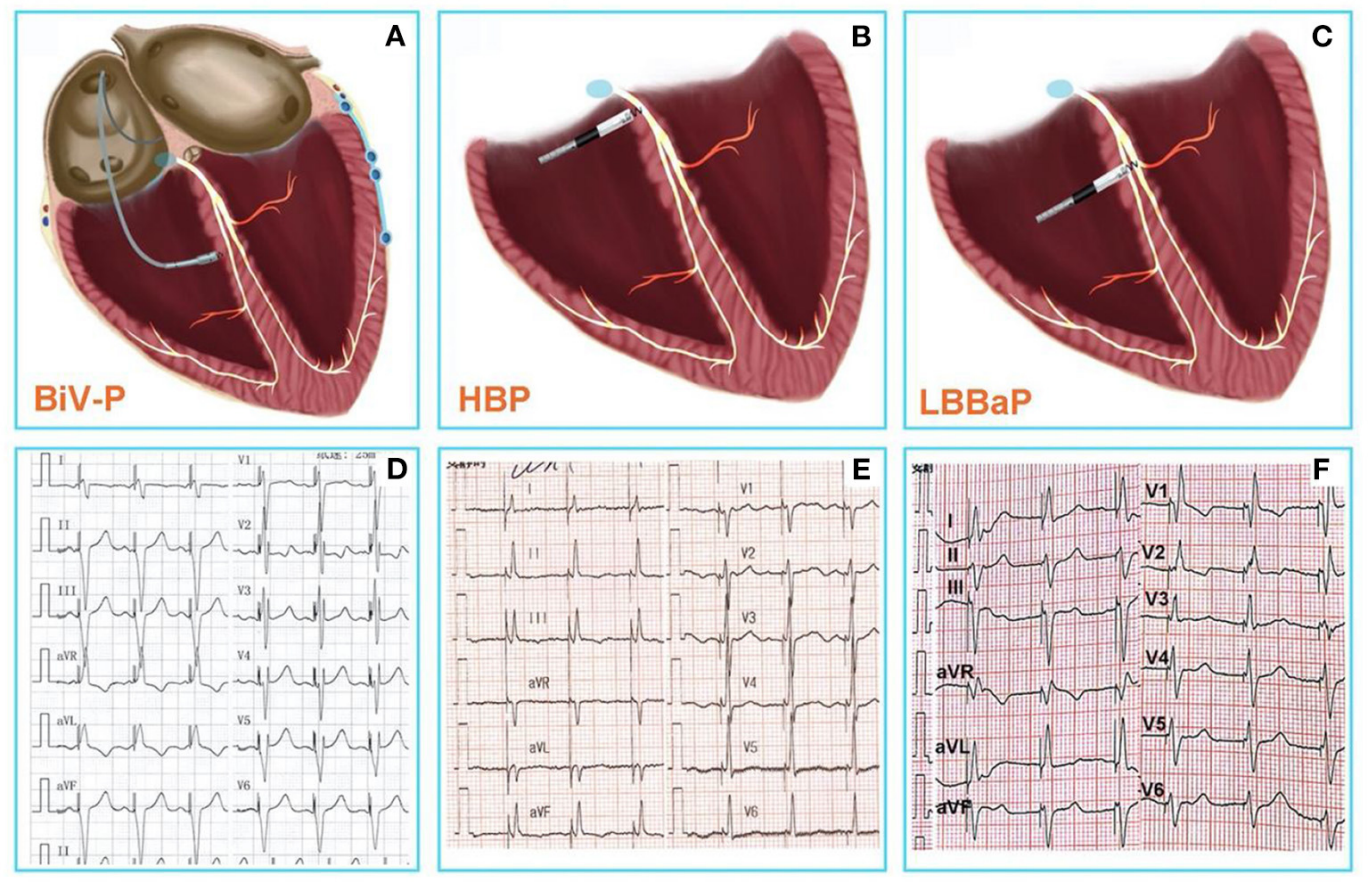
**(A)** Conventional CRT using BiV-p based on right ventricular pacing and coronary venous pacing. **(B)** HBP-the real physiological pacing modality directly activate the specialized conduction system. **(C)** LBBaP is pacing at more distal and deeper area than HBP, and pacing the left bundle branch region directly. **(D)** The 12-lead ECG after BiV-p, the paced QRS duration was 134 ± 15 ms (7). **(E)** The 12-lead ECG after HBP, the paced QRS duration was 103.8 ± 13 ms (8). **(F)** The 12-lead ECG after LBBaP, the paced QRS duration was 114.1 ± 10.7 ms (8). CRT, Cardiac resynchronization therapy; BiV-p, biventricular-pacing; HBP, His bundle pacing; LBBaP, left bundle branch area pacing; ECG, electrocardiogram.

A large number of studies have shown that the traditional BiV-CRT can effectively correct the asynchrony of electromechanical activity in heart failure patients, thereby

improving cardiac function and reducing the mortality of these patients (4–6) (Figures 1A,D). However, the electrical synchrony restored by traditional BiV is not physiological, as it is achieved by variable fusion of wavefronts propagating from the endocardium and epicardium (9). In addition, the failure rate range between 7.5 and 10% due to left ventricle scars and coronary vein stenosis or deformity such as diffcult coronary sinus access, tortuous and stenotic venous branches and tiny venous branches (10–13), and about 30–40% of patients do not respond to BiV-CRT due to lead instability, increased pacing thresholds, and phrenic nerve stimulation (14–16).

Therefore, physiological pacing techniques that directly activate the specialized conduction system are gaining increasing popularity (Figures 1B,E). Compared to BiV-CRT, HBP—the real physiological pacing modality first reported by Deshmukh et al. (2)—can achieve similar electrical synchronization and LVEF improvement (7). However, the poor sensing amplitude, the increase of pacing threshold over time, and the inability of the implantation site to cross the block site are the disadvantages associated with HBP, which limit the wide application of HBP to all patients with pacing and CRT indications (3, 17).

It is encouraging that a novel physiological pacing strategy, LBBaP that has emerged in recent years has significant advantages (18) (Figures 1C,F). Meanwhile, mounting evidence indicates that LBBaP appears to be an effective method for CRT, and is associated with improvements of symptoms and cardiac function (19–22). The result of latest, prospective, randomized study of LBBaP-CRT vs. BiV-CRT has shown that LBBaP-CRT could achieve better LVEF improvement than BiV-CRT in heart failure patients with non-ischemic cardiomyopathy and left bundle branch block (LBBB) (23). In comparison with BiV-CRT and HBP, LBBaP has a lower and more stable pacing capture threshold, a higher success rate of implantation, a shorter learning curve, and greater economic feasibility (24, 25). Non-etheless, LBBaP is not suitable for all heart failure patients with CRT indications. Accordingly, we conducted a literature review on the success rates, challenges, and troubleshooting of LBBaP in heart failure patients needing a CRT.

## The success rate of LBBaP-CRT

The success rates of LBBaP-CRT range from 81.1 to 98.1% in cases of patients with CRT indications in all 6 studies included in this review (Table 1) (9, 19–21, 26). The failure rate of BiV-CRT is high due to coronary vein stenosis or deformity (10) and about 30–40% of patients do not respond to BiV-CRT (14–16). HBP has the disadvantages of poor sensing amplitude, the increase of pacing threshold over time, and the inability of the implantation site to cross the block site (3, 17). Compared with HBP-CRT and BiV-CRT, LBBaP-CRT has a higher success rate of the implant and many advantages. For experienced doctors, the percentage of an LBB lead being implanted into an ideal area can reach more than 98% (26), whereas the success rates of an HBP lead and left ventricular lead being implanted into a targeted region are 92% and 90%, respectively (26, 27).

**Table 1:** Characteristics of studies included in the review.

**Reference**	**Design**	**Indication for CRT**	**Baseline QRS duration (ms)**	**LVEF (%)**	**Number of HF patients**	**Number of success**	**success rate (%)**	**Reasons of failure**	**Solution (Number of success)**	**Total success rate (%)**
Huang et al. (19)	Prospective, multicenter	Symptomatic heart failure with LVEF <50% with CLBBB,	168.6 ± 16.4	33 ± 7.4	63	61	96.8	Failure to capture the LBB	BiV-CRT (2)	100
Vijayaraman et al. (20)	Retrospective, observational, multi-center	Symptomatic heart failure with LVEF ≤ 50% with CLBBB,NYHA functional class II-IV	154 ± 32	32 ± 12	325	277	85.2	Inability to penetrate the septum	BiV-CRT (44)	98.8
Li et al. (21)	Prospective, observational, multi-center	Symptomatic heart failure with LVEF ≤ 35% with LBBB, and had received≥4 months GDMT	177.9 ± 18.8	29.3 ± 5.9	37	30	81.1	Failure to capture the LBB, Inability to penetrate the septum, VT induced	BiV-CRT (4)	91.9
Guo et al. (9)	Prospective, observational, single-center	LBBB defined by Strauss criteria, NYHA functional class II-IV with LVEF ≤ 35%	167.7 ± 14.9	30 ± 5	24	21	87.5	Inability to penetrate the septum	BiV-CRT (3)	100
Chen et al. (26)	Non-randomized, prospective, observational, multi-center	LBBB defined by Strauss criteria, symptomatic heart failure with LVEF ≤ 35%, NYHA functional class II-IV	180.12 ± 15.79	29.05 ± 5.09	49	48	98.1	Inability to penetrate the septum	BiV-CRT (1)	100

CRT, cardiac resynchronization therapy; LVEF, left ventricular ejection fraction; CLBBB, complete left bundle branch block; NYHA, New York Heart Association; LBBB, left bundle branch block; GDMT, guideline-directed medical therapy; HF, heart failure; LBB, left bundle branch; VT, ventricular tachycardia. BiV-CRT, biventricular cardiac resynchronization therapy.

## Challenges of LBB lead implantation

Although LBBaP-CRT has a high success rate of implantation, it is not suitable for all heart failure patients needing a CRT (7, 9–21, 26). Challenges of LBBaP in heart failure patients with cardiac electromechanical activity synchronization are described below (Table 1).

## Inability of screwing the pacing lead into the interventricular septum

The failure to screw the pacing lead into the interventricular septum may be the following:

(1) Myocardial fibrosis: Myocardial fibrosis is a disease characterized by cell proliferation and excess extracellular matrix deposition in the normal myocardial tissue (28). The main pathological manifestations of myocardial fibrosis are increased myocardial stiffness, decreased myocardial contractility, and decreased coronary blood flow reserve. LBBaP captures the left conduction system (including the trunk of the left bundle branch or its proximal branches) through transvenous transseptal pacing. Therefore, myocardial fibrosis results in the inability of the pacing lead to be screwed into the left bundle branch area, rendering patients non-suitable for correction of cardiac electromechanical activity synchronization.
(2) Interference by the septal tricuspid leaflet: If the location of pacing lead implantation is close to the tricuspid annulus (TVA), it will be difficult to implant the pacing lead into septum because of the interference of septal tricuspid leaflet. If successful in this location, the septal tricuspid leaflet will be pinned to the septum (29).
(3) Pacing lead is not coaxial with sheath: The coaxiality between the pacing lead and the sheath is an important factor for implantation of the LBB lead to the targeted region. It is difficult to screw the pacing lead into an LBB area accurately if the lead is not coaxial with the delivery sheath for some reasons, which causes ineffective conduction of force.
(4) The sheath is not perpendicular to the interventricular septum: The vertical angle between the C315HIS delivery sheath and the septum is another key factor for the successful implantation of LBB lead. In our experience, if the C315HIS delivery sheath is not perpendicular to the septum, it is not conducive to the effective conduction of force, thereby increasing the difficulty of penetrating the pacing lead throughInterference by the septal tricuspid leaflet the septum.
(5) A creased sheath: The C315HIS delivery sheath could be creased after repeated manipulation in complicated cases. This inevitably increases the resistance to the penetrating pacing lead, making it difficult to screw the pacing lead correctly.

## Failure to capture the LBB

The following are common reasons for being unable to capture LBB:

(1) Myocardial fibrosis: Some patients exhibit local myocardial fibrosis in the left bundle branch area. In these patients, even though the pacing lead can be successfully screwed in place, the LBB cannot be captured due to local myocardial fibrosis.
(2) Distal LBBB: Studies have shown that the majority of the complete LBBB blocks are located in the left-sided His fibers (left intrahisian) and the proximal left bundle branch (24). Tung et al. reported that 64% of the block sites were in the left proximal His-Purkinje conduction system (72% in the His bundle and 28% in the proximal left bundle branch) (25). LBBaP is transvenous transseptal pacing to capture the left conduction system (including the trunk of the left bundle branch and its proximal branches). Therefore, LBBaP cannot correct patients with complete LBBB block sites distal to the left bundle branch (30).
(3) Non-specific intraventricular conduction disturbance (IVCD): Non-specific IVCD is associated with conduction diseases within the ventricular wall (such as Purkinje fiber network or working myocardium), while its proximal conduction system (such as the His bundle and its major bundle branches) works normally (31). IVCD occurs in various cardiomyopathy and myocardial infarction. The American Heart Association/American College of Cardiology Foundation/Heart Rhythm Society (AHA/ACCF/HRS) recommendations, published in 2009, specify that non-specific IVCD in adults is defined by “a QRS duration >110 ms without meeting the criteria for RBBB or LBBB” (32). Similarly, LBBaP cannot restore cardiac electromechanical synchrony in heart failure patients with CRT indications combined with non-specific IVCD.
(4) Pacing lead did not reach the left bundle branch area: In the early days of LBBaP, less experienced doctors may have operated on a small number of cases, where less precise placement of the pacing lead into the left bundle branch area may occur, leading to failure to capture the LBB.
(5) Interventricular septal perforation: Chen et al. reported 5 failed LBBaP cases due to interventricular septal perforation. The pacing lead penetrated into the left ventricle after repeated attempts at different sites due to a thin or soft interventricular septum (33). Therefore, for heart failure patients with a thin or soft interventricular septum, interventricular septal perforation is a common cause of LBBaP failure.

## Enlargement of the right atrium or right ventricle

The right atrium or right ventricle can become enlarged significantly because of dilated cardiomyopathy, rheumatic valve disease or pulmonary heart disease, to name a few. The length of the C315HIS delivery sheath (Medtronic Inc) is 43 cm, which may not be adequate for an enlarged right atrium or right ventricle. The contact between the pacing lead and the interventricular septum is unstable, and the interventricular septum cannot be reached. As a result, the pacing lead cannot be screwed into the left bundle branch area (34).

## Troubleshooting of LBBaP-CRT

### Improve the success rate of LBBaP

**Figure 2:**
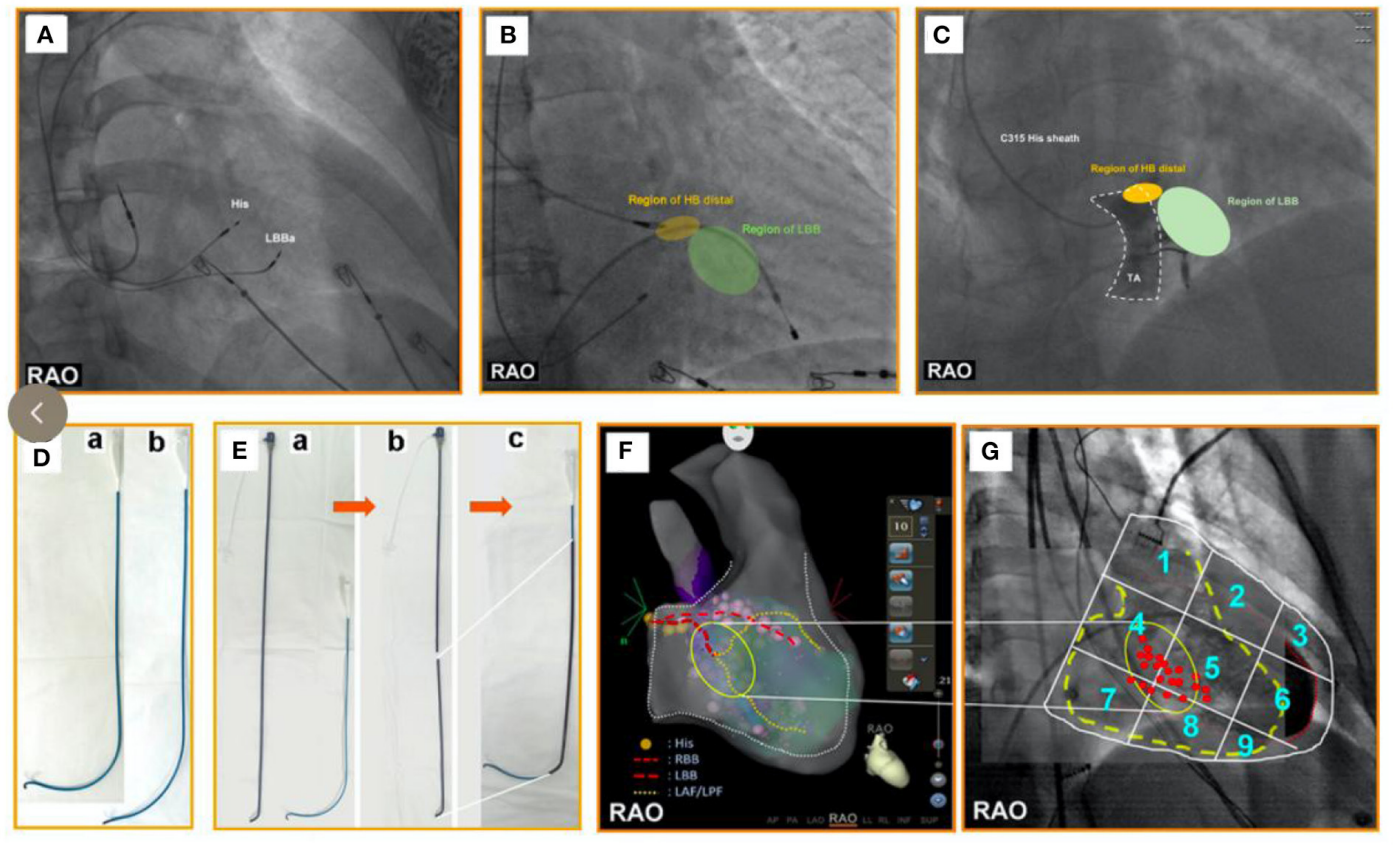
**(A)** One pacing lead is placed in the His-bundle region—an anatomical landmark. Another pacing lead is screwed into the LBBa. The position of the LBBaP is about 1–1.5 cm distal to the HBP lead position (i.e., the site of the first pacing lead) in the interventricular septum along the line connecting the HBP position and right ventricular apex from the RAO view. **(B)** The yellow area represents the HB distal region. The green area represents the LBB region that is regarded as an initial implant site of LBBaP. The region of LBB is ~1–1.5 cm distal to the region of HB in the interventricular septum along the line between the region of HB and the right ventricular apex in the right anterior oblique view. **(C)** The area surrounded by the white dotted lines represents TA. The yellow area represents the HB distal region that is close to the TA summit. The green area represents the LBB region that is regarded as an initial implant site of LBBaP. From the RAO view, the region of LBB is ~1–1.5 cm distal to the region of HB in the interventricular septum along the line between the region of HB and the right ventricular apex. **(D)** a. The C315HIS delivery sheath with regular curvature; b. The C315HIS delivery sheath with increased curvature. **(E)** a. Atrial septal puncture sheath (8.5F, T1, 62 cm) and C315HIS delivery sheath; b. Severed atrial septal puncture sheath (8.5F, T1, 62 cm); c. C315HIS delivery sheath coated part of atrial septal puncture sheath. **(F)** His bundle and LBB are tagged under three-dimensional mapping, followed by pacing lead positioning into the LBBa. **(G)** We numbered the 3 × 3 partitions from 1 to 9 in the RAO fluoroscopic image of the ventricle. Most successful initial implant sites were found at the junction of the partition zones “4/5/7/8” with more points located in zones “4 and 5”. RAO, right anterior oblique; LBBa, left bundle branch area; HB, His bundle; LBB, left bundle branch; TA, tricuspid annulus; HBP, His bundle pacing.

For failed cases of LBBaP-CRT, the following methods can be used to improve the success rate:

(1) The dual-lead method (Figure 2A) (35, 36): First, a pacing lead is placed in the His-bundle region—an anatomical landmark. Second, another pacing lead is screwed into the left bundle branch area. The position of the LBBaP is about 1–1.5 cm distal to the HBP lead position (i.e., the site of the first pacing lead) in the interventricular septum along the line connecting the HBP position and the right ventricular apex from the right anterior oblique view (Table 2). The appropriate extension this distance may be appropriate for heart failure patients with enlarged right ventricles. Third, the first pacing lead was moved to right atrium for atrial pacing. The dual-lead method is available to all patients; it can improve the success rate of the LBBaP significantly, especially in complicated cases.
(2) His potential mapping (Figure 2B): A pacing lead is placed in the His-bundle region *via* the C315HIS delivery sheath by identifying His potential. Then, the pacing lead is advanced to 1–1.5 cm distal to the previous position in the interventricular septum along the line between the previous position and right ventricular apex in the right anterior oblique view. It should be noted that a quadripolar mapping catheter is an alternative to the pacing lead to identify His potential (37).
(3) TVA visualization (Figure 2C): Visualization of the TVA with a contrast medium can help determine the pacing lead tip location. This technique facilitates the implantation of the pacing lead during the procedure of LBBaP. In addition, this approach can significantly reduce the operative time and fluoroscopy time, as well as the time for pacing lead repositioning attempts (29). At last, operators can avoid the location near the TVA with the help of this technique, which may avoid the entrapment of septal tricuspid leaflet. However, it should be noted that this method is not suitable for patients with a contrast medium allergy and renal insufficiency.
(4) Reshaping the delivery sheath (Figure 2D): For patients with an unstable contact between the C315HIS delivery sheath and the interventricular septum owing to enlargement of the right ventricle or persistent left superior vena cava, the C315HIS delivery sheath could be reshaped. The increased curvature enhances the stability of the interface between the delivery sheath and the ventricular wall, making it easier to screw the pacing lead into the left bundle branch area (38).
(5) Using deflectable delivery sheath: If available, the deflectable delivery sheath included C304 HIS sheath and Agilis HisPro sheath is also a prety choice for patients with enlargement of the right ventricle (37, 39). The mechanism of this approach is similar to tip 5.
(6) “Sheath-in-sheath” technique (Figure 2E): The large bending angle of the sheath will be gradually decreased after the inner sheath and the outer sheath are assembled. For patients with an unstable contact between the C315HIS delivery sheath and the interventricular septum due to right atrium enlargement, the “sheath-in-sheath” technique (C315 HIS delivery sheath as the inner sheath and CS sheath) can be used to strengthen the stability of the interface to facilitate the placement of the pacing lead into the left bundle branch area (39). Under certain special circumstances, the part of atrial septal puncture sheath can be regarded as the outer sheath and it should be noted that you need to be careful when removing the atrial septal puncture sheath using scissors.
(7) Three-dimensional mapping (Figure 2F): Vijayaraman P et al. successfully tagged the His bundle and left bundle branch aera by three-dimensional mapping, followed by LBBaP. Three dimensional mapping not only lowers the learning curve for beginners but also facilitates the evaluation of pacing lead depth during the procedure (39). Furthermore, LBBP can be performed successfully in patients with left ventricular hypertrophy, ventricular septal defect repair and during AV node ablation with the help of three-dimensional mapping (40).
(8) Simplified nine-partition method (Figure 2G): An electrophysiological recording system is usually required for the traditional LBBaP implantation method, which is expensive and not readily available in all centers (43, 44). Besides, it is not easy to find a suitable initial site for fixation. Although the LBBaP definition and implantation procedure have been well described by Huang et al. (36), it is difficult to grasp by less experienced operators. A simplified “nine-partition method” was first introduced by Zhang et al. for physiological LBBaP (41). In this method, the most successful initial implant sites were found at the junction of the partition zones “4/5/7/8” with more points located in zones “4 and 5”. LBBaP can be performed successfully by screwing the pacing lead into the “4/5” partitions. Compared with the conventional approach, the simplified “nine-partition method” not only saves time but also eliminates the requirement for expensive electrophysiological recording devices for some centers. Furthermore, this approach is suitable for all patients and has contributed to improved success rates of LBBaP by less experienced operators.
(9) Intracardiac echocardiography (ICE): The key step of the LBBaP process is to screw the pacing lead into the left bundle branch area. Currently, we perform this procedure by relying on X-ray images and intracavitary electrocardiograms combined with the morphology of the electrocardiogram after pacing. However, it is difficult to determine the depth of the pacing lead within the myocardium under X-ray fluoroscopy, which exacerbates the difficulties in surgery as well as the risk of perforation (45–48). The position of the pacing lead in the cardiac chamber and the depth of the pacing lead into the myocardium can be displayed clearly by ICE in real time. Therefore, under “direct view” of ICE, the operator can accurately screw the pacing lead into the left bundle branch aera, while preventing the pacing lead from penetrating the ventricular septum and entering the left ventricle (49). This approach is suitable for all patients, and the success rate and safety of complicated operations can be improved (42).

**Table 2:** The strategy of improving the success rate of LBBaP.

**Solving strategy**	**Indication**	**Characteristic**	**Implant success (%)**	**References**
The dual-lead method	Almost all	Expensive	Unknown	Zhang et al. (35)
His potential mapping	Almost all	Simple and expensive	Unknown	Vijayaraman et al. (37)
TVA visualization	Majority of	Simple and economical	96.7	Hua et al. (29)
Reshaping of delivery sheath	Right ventricle enlarged	Simple and economical	Unknown	Prolič Kalinšek et al. (38)
Using deflectable delivery sheath	Right ventricle enlarged	Simple and expensive	Unknown	Vijayaraman et al. (37) and Huang et al. (39)
The “sheath-in-sheath” technique	Right atrium enlarged	Simple and economical	Unknown	Huang et al. (39)
Three dimensional mapping	Almost all	Expensive	Unknown	Vijayaraman et al. (40)
The simplified “nine-partition method”	Almost all	Simple and economical	92.9	Zhang et al. (41)
ICE	Almost all	Expensive	Unknown	Vijayaraman et al. (42)

TVA, tricuspid valve annulus; ICE, intracardiac echocardiography.

### Adjust to HBP-CRT

Heart failure patients with an unsuccessful LBBaP should receive a different treatment strategy. HBP is the most physiological pacing method (2) that contributes to observable improvement in cardiac function (7). HBP-CRT, therefore, serves as an alternative to LBBaP-failed patients. However, the poor sensing amplitude, the increase of pacing threshold over time, and the inability of the implantation site to cross the block site are the disadvantages associated with HBP, which limit the wide application of HBP to all patients with pacing and CRT indications (3, 17).

### Adjust to BIV-CRT

BiV-CRT is a traditional CRT technique that is recommended as the primary treatment for heart failure patients with reduced LVEF and asynchrony of cardiac electromechanical activity. For heart failure patients after an unsuccessful LBBaP, BiV-CRT is an alternative therapy. After adjusting to BiV-CRT, the success rate of CRT can be improved significantly (91.9–100%) in some cases (9, 19–21, 26). Hence, BiV-CRT is still an irreplaceable treatment for specific patients. However, the electrical synchrony restored by traditional BiV is not physiological, as it is achieved by variable fusion of wavefronts propagating from the endocardium and epicardium (9). In addition, the failure rate is high due to coronary vein stenosis or deformity (10) and about 30–40% of patients do not respond to BiV-CRT (14–16).

For the past few years, stylet-driven pacing leads (SDL) has been reported to be safe and feasible during the procedure of LBBaP (50). However, the clinical data and experience about SDL are limited, and even less in the challenging patient such as heart failure. With the increasing clinical application of SDL in LBBaP and continuous accumulation of experience, innovative operators will summarize very excellent experience.

LBBaP is an effective pacing strategy for heart failure patients with CRT indications and has a high overall success rate. The following patients may be candidates for LBBaP-CRT: (1) heart failure patients presented sinus rhythm and complete LBBB morphology, with LVEF ≤ 35% and New York Heart Association (NYHA) functional class III to IV. (2) patients who have a significantc proportion of right ventricular pacing and moderate LVEF. (3) patients with long-standing persistent atrial fibrillation combined with heart failure requiring atrioventricular nodal ablation. (4) patinets with conventional right ventricular pacing-induced cardiomyopathy. However, LBBaP is not suitable for all heart failure patients needing a CRT such as myocardial fibrosis, distal LBBB or non-specific IVCD.

In this review, We summarized 9 practical methods and strategies that could improve the success rate of LBBaP-CRT. Taken together, LBBaP is one of the important technologies to achieve CRT effectively. LBBaP, HBP, and BiV-CRT can complement each other and jointly improve the success rate of CRT in patients with heart failure.

## Author contributions

CM and JZ put forward the idea and the structure of the article. YZ searched the literature, collected the data, and wrote the manuscript. JZ finished figures. ZW provided some images. JZ, YS, and MC reviewed and revised the manuscript. All authors read and approved the final manuscript.
